# *Sagaratremaeugari* (Tubangui and Masilungan, 1936) (Digenea: Liolopidae) bridges a gap in liolopid diversity in sea snakes

**DOI:** 10.3389/fvets.2026.1895052

**Published:** 2026-08-18

**Authors:** Nannaphat Suwannarat, Alexis Ribas Salvador, Carmen Gilardoni, Pongsak Laudee, Srisupaph Poonlaphdecha

**Affiliations:** 1 Program in Fishery Science and Aquatic Resources, Department of Agricultural Technology, King Mongkut’s Institute of Technology Ladkrabang, Prince of Chumphon Campus, Chumphon, Thailand; 2 Section of Parasitology, Department of Biology, Healthcare and Environment, Faculty of Pharmacy and Food Science, University of Barcelona, Barcelona, Spain; 3 Institute of Research in Biodiversity (IRBio), University of Barcelona (UB), Barcelona, Spain; 4 Faculty of Innovative Agriculture, Fisheries and Food, Prince of Songkla University, Surat Thani Campus, Surat Thani, Thailand

**Keywords:** Acrochordidae, *Acrochordus*, helminth parasite, little file snake, Thailand, trematode

## Abstract

**Introduction:**

The family Liolopidae comprises digenean trematodes parasitizing amphibians and reptiles, although the life cycles and evolutionary history of several taxa remain poorly understood. Species of *Sagaratrema* are primarily associated with marine elapid snakes from the Indo-West Pacific region; however, *Sagaratrema eugari* is exceptional because it has previously been reported from non-marine snake hosts. This study aimed to identify *S. eugari* from the little file snake, *Acrochordus granulatus*, and to provide its first molecular characterization and phylogenetic assessment.

**Methods:**

During parasitological surveys conducted in the Gulf of Thailand (Surat Thani and Chumphon provinces), five specimens of *A. granulatus* were examined for helminths. Recovered liolopid trematodes were identified based on morphology and characterized using based on *18S*, ITS2, *28S* rDNA, and *COI* mtDNA sequences. Phylogenetic analyses were performed to determine the evolutionary position of the species.

**Results:**

Liolopid trematodes were recovered from all three snakes examined in Surat Thani Province, whereas no infections were detected in the two specimens from Chumphon Province. Morphological observations were consistent with the original description of *S. eugari* and with the diagnostic features of the genus *Sagaratrema*. Molecular data provided the first genetic characterization of the species, and phylogenetic analyses confirmed its placement within *Sagaratrema. Acrochordus* granulatus is identified as a definitive host for *S. eugari*.

**Discussion:**

This study resolves the previously unknown phylogenetic position of *S. eugari* and expands the known host range of *Sagaratrema* beyond hydrophiine sea snakes. The findings suggest that the transmission ecology of *S. eugari* may involve interactions among mangrove, estuarine, and marine habitats. However, additional sampling across the parasite’s geographic range is required to clarify its host associations and elucidate its complete life cycle.

## 1 Introduction

The family Liolopidae Odhner, 1912 comprises a small group of digenean trematodes whose adult stages parasitize aquatic reptiles and amphibians. The family currently includes seven genera with distinct host associations. The genus *Liolope* Cohn, 1902 infects amphibians, particularly giant salamanders in East Asia, as well as freshwater turtles in Gabon. *Helicotrema* Odhner, 1912 occurs in reptiles such as iguanas, freshwater turtles, and tortoises in the Neotropics, whereas *Dracovermis* Brooks and Overstreet, 1978 and *Ngubuvangandu* Dutton and Bullard, 2026 parasitize crocodilians. The genus *Harmotrema* Nicoll, 1914 includes species infecting snakes, whereas *Paraharmotrema* Dutton and Bullard, 2022 is known from freshwater pelomedusid turtles in southeastern Africa reviewed by Niewiadomska (1), De Silva et al. (2). More recently, the genus *Sagaratrema* De Silva, Pathirana, Rajapakse and Martin, 2026 was established to accommodate liolopid trematodes infecting Indo-West Pacific marine snakes (2). Currently, *Sagaratrema* comprises six recognized species [From De Silva et al. (2)]: *Sagaratrema laticaudae* (Yamaguti, 1933), *Sagaratrema eugari* (Tubangui and Masilungan, 1936), *Sagaratrema indicum* (Chattopadhyaya, 1970), *Sagaratrema linguiforme* (Wang, 1987), *Sagaratrema rajakarunae*, and *Sagaratrema rajapaksei*. Four of these species were originally described in *Harmotrema* and were subsequently transferred following the establishment of *Sagaratrema* (2). Members of this genus are primarily associated with hydrophiine sea snakes and sea kraits, suggesting that their transmission cycles are largely marine. It has therefore been proposed that at least some species complete fully marine life cycles; however, the identity of their intermediate hosts remains unknown. Based on the ecology of their definitive hosts and comparisons with related digeneans, marine gastropods and fishes have been suggested as potential first and second intermediate hosts, respectively (2).

Within the genus, *S. eugari* is exceptional in its host range. Unlike most congeners, which infect marine elapids, *S. eugari* was originally described from the terrestrial Philippine cobra (*Naja philippinensis* Taylor, 1922) on Luzon Island, Philippines (3), and was subsequently reported from the Southeast Asian bockadam *Cerberus rynchops* (Schneider, 1799) on Mindanao Island, Philippines (4), a semi-aquatic species inhabiting marine, estuarine, and freshwater environments (5). However, no subsequent records of this trematode have been reported in the literature. This unusual host association suggests that the life cycle of *S. eugari* may differ from that of other species within the genus, or that previously reported hosts may represent accidental infections; however, its transmission pathways and intermediate hosts remain unknown (2).

In addition, the phylogenetic position of *S. eugari* within *Sagaratrema* has not yet been resolved, limiting our understanding of its evolutionary significance. Ribosomal DNA (rDNA) is highly repetitive and contains variable regions (ITS) that are useful for species identification, alternating with conserved regions (*18S*, *28S*) that are suitable for phylogenetic studies (6). In recent years, these markers have been widely used to study digeneans, e.g., (7, 8). The *COI* gene of mitochondrial DNA (mtDNA) holds significant value for species differentiation within the same genus and is increasingly utilized in diagnostics, providing precise genetic markers for accurate taxonomic identification (9).

The little file snake, *Acrochordus granulatus* (Schneider, 1799) (Acrochordidae Bonaparte, 1840), is widely distributed from the northwestern coast of India to the Solomon Islands and occupies a broad range of habitats, including freshwater systems, mangroves, estuaries, reefs, and coastal marine environments, as reviewed by Sanders et al. (10). In contrast, the two other extant species exhibit marked ecological differences: *Acrochordus javanicus* Hornstedt, 1787 is primarily associated with freshwater and occasionally brackish habitats in Southeast Asia, whereas *Acrochordus arafurae* McDowell, 1979 is restricted to freshwater systems in northern Australia and southern New Guinea (10). These ecological differences suggest a freshwater origin for the genus, followed by subsequent diversification into brackish and marine environments.

Previous studies on helminths infecting *A. granulatus* are scarce. Early surveys from the Arabian Sea reported several digenean species, including *Simhatrema simhai* Chattopadhyaya, 1971, *Acanthostomum cherysdrusi* (Chattopadhyaya, 1971), and *Gogatea mehri* Mehra, 1947 (11). Other records include nematodes identified as *Goezia* sp. from Australia (12). More recently, De Silva et al. (2) examined seven specimens of *A. granulatus* from Sri Lanka but did not detect liolopid trematodes. To date, liolopid trematodes have not previously been reported from the little file snake.

During parasitological surveys of marine and estuarine vertebrates in the Gulf of Thailand (13), specimens of *A. granulatus* were examined, and a liolopid trematode was recovered and identified as *S. eugari*. Given the limited knowledge of this trematode, particularly regarding its host associations, life cycle, and phylogenic position, the present study aims to address these gaps through an integrative approach combining morphological observations with molecular characterization based on *18S*, ITS2, *28S* rDNA, and *COI* mtDNA sequences. These data were used to assess the phylogenetic placement of *S. eugari* within *Sagaratrema* and the family Liolopidae, thereby contributing to a better understanding of its biology and evolutionary relationships.

## 2 Materials and methods

### 2.1 Sampling

In April 2025, four dead specimens of *A. granulatus* were opportunistically collected from Phum Riang Canal, Chaiya District, Surat Thani Province, Thailand (9.37447°N, 99.26798°E), a coastal waterway connected to the Gulf of Thailand. The specimens were transported to the laboratory and kept refrigerated prior to dissection, which was conducted approximately 48 h after collection. Although liolopid trematodes were recovered, the parasites were poorly preserved and exhibited deformation and loss of diagnostic morphological characters, precluding reliable identification and morphometric analyses. Because the time elapsed since host death and the duration of exposure to high ambient temperatures prior to collection could not be determined, these specimens were excluded from the present study. The locality is an estuarine environment connected to the Gulf of Thailand and includes mangrove-associated habitats.

Subsequently, in August 2025, the same locality was revisited, and five additional specimens of *A. granulatus* were collected. Three specimens were examined immediately in a field laboratory, whereas the remaining two were dissected later. However, the latter specimens showed a similar degree of parasite degradation, preventing reliable morphological examination, although trematode infection was detected. Consequently, only the three specimens examined immediately after collection were included in the present study. In addition, two specimens were obtained near Koh Kai Island, Pathio District, Chumphon Province, Thailand (10.70587°N, 99.40696°E), a shallow coastal marine locality. A total of 11 specimens of *A. granulatus* were obtained during the study, of which five were included in the parasitological analyses.

All specimens were collected from local fishermen as incidental bycatch. No animals were intentionally captured or euthanized for the proposes of this study. Because the little file snake has no commercial value, individuals captured in fishing nets are typically killed, removed from the nets, and discarded by local fishermen. Host identification was based on morphological characters following (10). The examined specimens ranged from 405 to 770 mm in total length. This study was reviewed and approved by the Animal Care and Use Committee of King Mongkut’s Institute of Technology Ladkrabang, Thailand, and was conducted in accordance with local legislation and institutional requirements.

### 2.2 Morphological study

Specimens from Surat Thani Province were examined for helminths immediately after capture in a field laboratory, whereas specimens from Chumphon Province were examined at the laboratory of King Mongkut’s Institute of Technology Ladkrabang, Chumphon Campus. The entire digestive tract was removed, opened longitudinally, and examined under a stereomicroscope in saline solution. Specimens were heat-killed in near-boiling saline and immediately fixed in 70% ethanol, stained with Semichon’s acetocarmine, dehydrated through a graded ethanol series, cleared in xylene, and mounted in Canada balsam. A total of 10 stained gravid specimens were examined morphologically. Observations and measurements were made using a Leica DMLB microscope equipped with a digital camera. Measurements were obtained from mounted specimens. All measurements are provided in μm unless otherwise stated, as mean values followed by ranges in parentheses. All examined specimens were deposited in the Museum of Natural Sciences of Barcelona under collection numbers MZB 2026-0206 to MZB 2026-0215.

### 2.3 Molecular study

Molecular analyses were conducted on two specimens using rDNA (*18S*, ITS2, and *28S*) and mtDNA (*COI*) markers, this limited number did not allow to explore the intraspecific genetic variation. Genomic DNA was extracted using the DNeasy Blood and Tissue Kit (Qiagen) following the manufacturer’s instructions. The *18S*, ITS2, and *28S* regions of the rDNA, as well as the *COI* region of the mtDNA, were amplified by PCR. The PCR amplifications were performed in a total reaction volume of 25 μL containing 1x Green GoTaq^®^ Reaction Buffer (1.5 mM MgCl_2_ for rDNA markers and 2 mM MgCl_2_ for the mtDNA marker), 0.2 mM each dNTP, 1 μM of each primer, and 5 U/μL GoTaq^®^ DNA Polymerase (Promega). The *18S* region was amplified using the primers 18S F (5-ATCCGAAGTAATGGTTAAGAGGG-3) and 18S R (5-ACCTACGGAAACCTTGTTACG-3) (14); the ITS2 region using ITS2 F (5-GCTCGTGTGTCGATGAAGAG-3) and ITS2 R (5-AGGCTTCGGTGCTGGGCT-3); and the *28S* region using the primers 28S F (5-GTGAATACCCGCTGAACTTAAGC-3) and 28S R (5-TCTCCTTGGTCCGTGTTTCAA-3) (14). For the rDNA markers, cycling conditions consisted of an initial denaturation at 94 °C for 5 min, followed by 40 cycles of 30 s at 94 °C, 30 s at 52 °C for *18S* and *28S* or 56 °C for ITS2, and 2 min at 72 °C, with a final extension step of 10 min at 72 °C. The *COI* region was amplified using the primers Dig_cox1F (5-ATGATWTTYTTYTTYYTDATGCC-3) and Dig_cox1R (5-TCNGGRTGHCCRAARAAYCAAAA-3) (15). Cycling conditions consisted of an initial denaturation at 94 °C for 2 min, followed by five cycles of 30 s at 94 °C, 40 s at 45 °C, and 1 min at 72 °C, followed by 35 cycles of 30 s at 94 °C, 40 s at 50 °C, and 1 min at 72 °C, with a final extension step of 10 min at 72 °C (16). Amplified PCR products were separated electrophoretically on a 1% (w/v) agarose gel stained with Green Gel. Negative controls were included in all PCR assays to detect potential contamination. PCR products were purified prior to sequencing using ExoSAP-IT Express (Life Technologies), an enzymatic cleanup method that removes residual primers and unincorporated dNTPs, following the manufacturer’s protocol; and sequenced, and all sequences were deposited in GenBank (*18S* PZ406322-PZ406323, ITS2 PZ406320-PZ406321, *28S* PZ406318-PZ406319, and *COI* PZ405784-PZ405785). Sequencing was performed by the Genomics Unit of the Scientific and Technological Centers (CCiTUB), University of Barcelona.

### 2.4 Phylogenetic analysis

Two phylogenetic trees were constructed for members of the family Liolopidae: one based on ITS2 sequences and another based solely on partial *28S* rDNA sequences (see species and outgroup data in Table 1). For ITS2 tree, *Cotylurus cornutus* was selected as an outgroup because it belongs to Diplostomidae, a family closer to Liolopidae. The *28S* tree was built according to De Silva et al. (2), the most current phylogeny for Liolopidae. Sequence alignments were generated using MAFFT (version 7) (available at http://www.ebi.ac.uk/Tools/msa/mafft/) and MEGA X (17). Phylogenetic and molecular evolutionary analyses were performed using the maximum-likelihood (ML) method implemented in MEGA X. The evolutionary model that best fit each dataset was determined using jModelTest 2.1.1 (18), with model selection based on the Akaike Information Criterion (AIC). The general time-reversible model with gamma-distributed rate variation among sites and a proportion of invariant sites (GTR + G + I) was selected as the best-fit model for the *28S* dataset, whereas the Kimura two-parameter model with invariant sites (K2 + I) was selected for the ITS2 dataset. The initial tree for this heuristic search was automatically selected by evaluating and comparing the log-likelihood values of a Neighbor-Joining (NJ) tree (constructed from a pairwise p-distance matrix) and a Maximum Parsimony (MP) tree (optimized over 10 independent searches with random starting topologies). To search the tree space and optimize branch lengths, the Nearest-Neighbor-Interchange (NNI) branch-swapping algorithm was employed as the primary heuristic method. Branch support was assessed using ultrafast bootstrap analysis with 1,000 replicates (19). Bootstrap support values are shown next to the corresponding branches. The *28S* dataset comprised 29 sequences with a total of 723 positions in the final alignment, whereas the ITS2 dataset included eight sequences with 362 positions. Positions containing gaps and missing data were removed using the complete deletion option. Finally, an unrooted Neighbor-Joining (NJ) tree was constructed in MEGA X using available *COI* sequences of *Sagaratrema* spp. retrieved from GenBank together with the *COI* sequences generated in the present study (Table 1). Because only a limited number of *COI* sequences are currently available for *Sagaratrema* spp., this analysis was used to visualize genetic relationships among the available sequences.

**Table 1:** Collection data and GenBank accession numbers for Liolopidae species included in the phylogenetic analyses.

Species	Stage	Host	*28S*	ITS2	*COI*	Reference
*Sagaratrema rajakarunae*	Adult	*Hydrophis cyanocinctus*	PX981821-22	PX981825	PX979758	(2)
*Sagaratrema rajapacksei*	Adult	*Hydrophis schistosus*	—	PX981824	PX979756-57	(2)
*Sagaratrema laticaudae*	Adult	*Laticauda laticaudata*	OL413009	—	—	(22)
*Sagaratrema rajakarunae*	Adult	*Hydrophis schistosus*	PX981822	—	—	(2)
*Sagaratrema indicum*	Adult	*Hydrophis schistosus*	PX981820	PX981823	PX979754-55	(2)
***Sagaratrema eugari***	**Adult**	***Acrochordus granulatus***	**PZ406318-19**	**PZ406320-21**	**PZ405784-85**	**Present study**
*Liolope copulans*	Cercaria	*Semisulcospira libertina*	AB551568	—	—	(23)
*Dracovermis occidentalis*	Adult	*Alligator mississippiensis*	PQ186364	PQ186369	—	(24)
*Paraharmotrema karinganiense*	Adult	*Pelomedusa galeata, Pelusios subniger*	OL413003	OL413008	—	(22)
*Ngubuvangandu francoisjacobsi*	Adult	*Crocodylus niloticus*	PX576118	—	—	(25)
Outgroup						
*Cotylurus cornutus*	Metacercaria	*Lithoglyphus naticoides, Stagnicola palustris*	PX457622	PX457652	—	(26)

Individuals studied in the present study are in bold.

## 3 Results

All three little file snakes examined from the Surat Thani locality were parasitized, with 24, 17, and 7 worms recovered per host, respectively. Specimens collected in April were excluded from all parasitological analyses because the recovered trematodes were poorly preserved and exhibited deformation and loss of diagnostic morphological characters. In addition, reliable assessment of parasite abundance was not possible because some trematodes may have been overlooked. The two individuals examined from Chumphon Province were negative for trematode infection.

**Figure 1:**
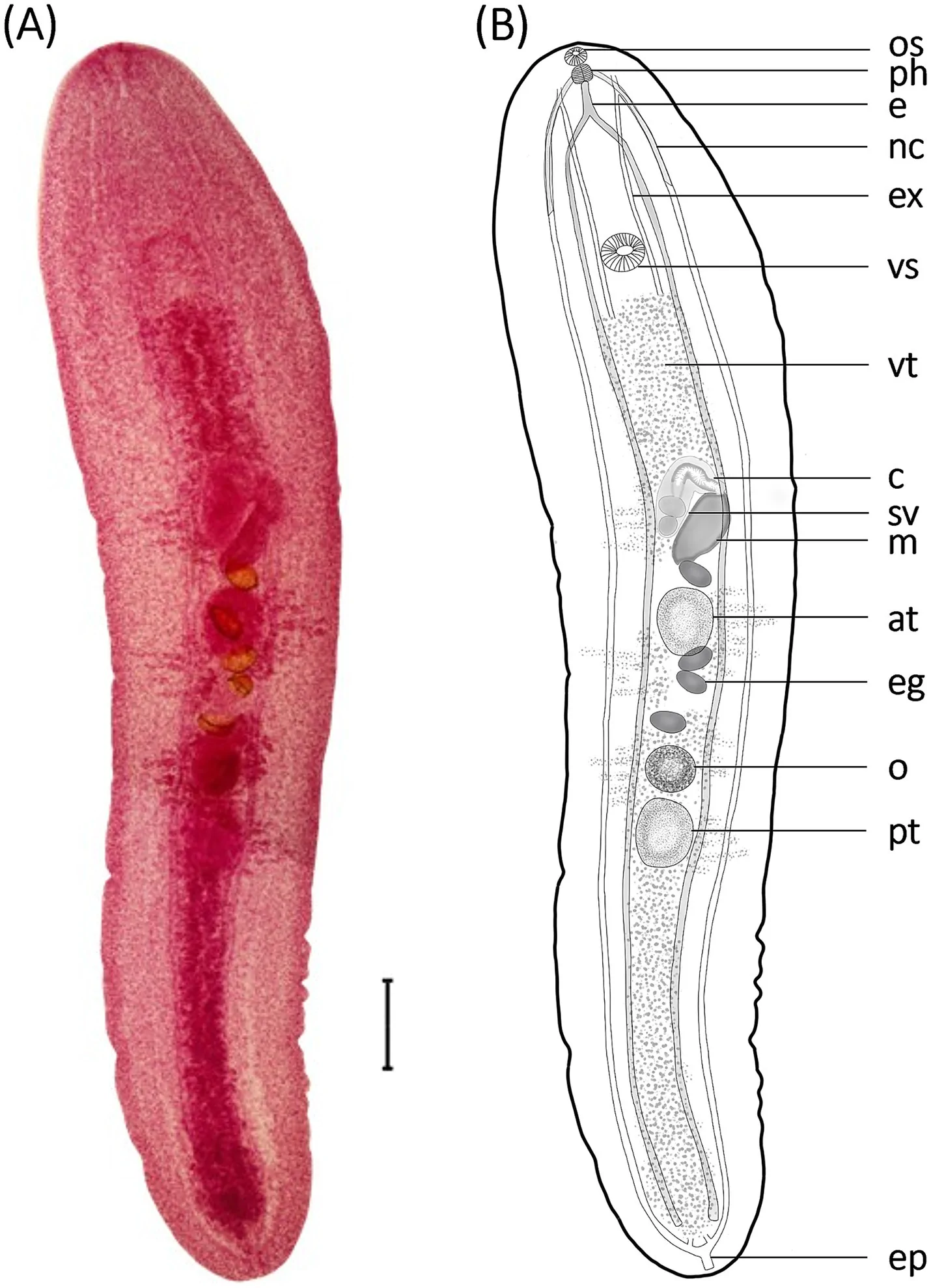
*Sagaratrema eugari* (Digenea: Liolopidae) collected from *Acrochordus granulatus* from Thailand. **(A)** Photomicrograph of a gravid specimen (ventral view). Scale bar = 200 μm. **(B)** Line drawing of the same specimen showing the main structures. Oral sucker (os), pharynx (ph), esophagus (e), nerve cord (nc), excretory system (ex), ventral sucker (vs), vitellarium (vt), cirrus (c), metraterm (m), seminal vesicle (sv), anterior testis (at), egg (eg), ovary (o), posterior testis (pt), and excretory pore (ep).

A total of 10 gravid specimens were examined morphologically and measured. The examined specimens agree with the original description of *S. eugari* (3), as well as the dichotomous identification key of (2). Body elongated and rounded at both extremities, 3,338 (2,914–3,677) long by 756 (627–974) wide at the level just posterior to the ventral sucker; body length/width ratio 4.4 (3.8–4.6). Tegument unarmed. Oral sucker subterminal, 71 (50–87) long by 74 (60–81) wide. Prepharynx absent. Pharynx oval and muscular, 69 (62–79) long by 65 (61–71) wide. Esophagus elongated, 85 (60–116). Intestinal ceca extending close to the median line, reaching near the posterior extremity. Ventral sucker small and rounded, 116 (101–132) long by 126 (113–140) wide, located in the anterior third of the body, 601 (462–707) from the anterior end, representing 18% (16–19%) of body length. Testes two, tandem, postequatorial, spherical to oval, separated by 313 (216–378). Anterior testis 189 (164–226) long by 193 (139–253) wide; posterior testis 213 (175–252) long by 194 (151–251) wide. Cirrus sac large, oval, preequatorial, lying obliquely between the intestinal ceca, a short distance anterior to the anterior testis, 274 (226–399) long by 139 (103–174) wide; enclosing a large seminal vesicle, pars prostatica, and an eversible cirrus. Cirrus densely covered with minute spine-like structures. Ejaculatory duct long, thick-walled, 118 (92–201) long by 59 (42–81) wide. Seminal vesicle 170 (110–289) long by 102 (81–131) wide. Ovary located between the testes, spherical to oval, close to or contiguous with the posterior testis, 182 (161–212) long by 161 (140–212) wide. Laurer’s canal present, opening dorsally at the level of the ovary. Vitellarium composed of small compacted follicles, with the anterior extent varying among individuals, reaching the level of or extending slightly anterior to the level of the ventral sucker, and extending posteriorly to the ends of the ceca. Vitellarium is mainly confined to the intercecal region, except in the zone between the cirrus sac and the posterior testis, where additional lateral patches of vitelline follicles occur. These patches are asymmetrically distributed, varying in both number (0–3 per side) and position between the left and right sides. Posterior to the testes, the vitelline fields are restricted to the intracecal space and never reach the excretory system. Uterus very short, leading to a well-developed metraterm, 163 (108–217) long by 87 (37–141) wide. Eggs few (2–14), oval, yellowish, 102 (84–116) long by 61 (45–71) wide (*n* = 70) (Figure 1).

**Figure 2:**
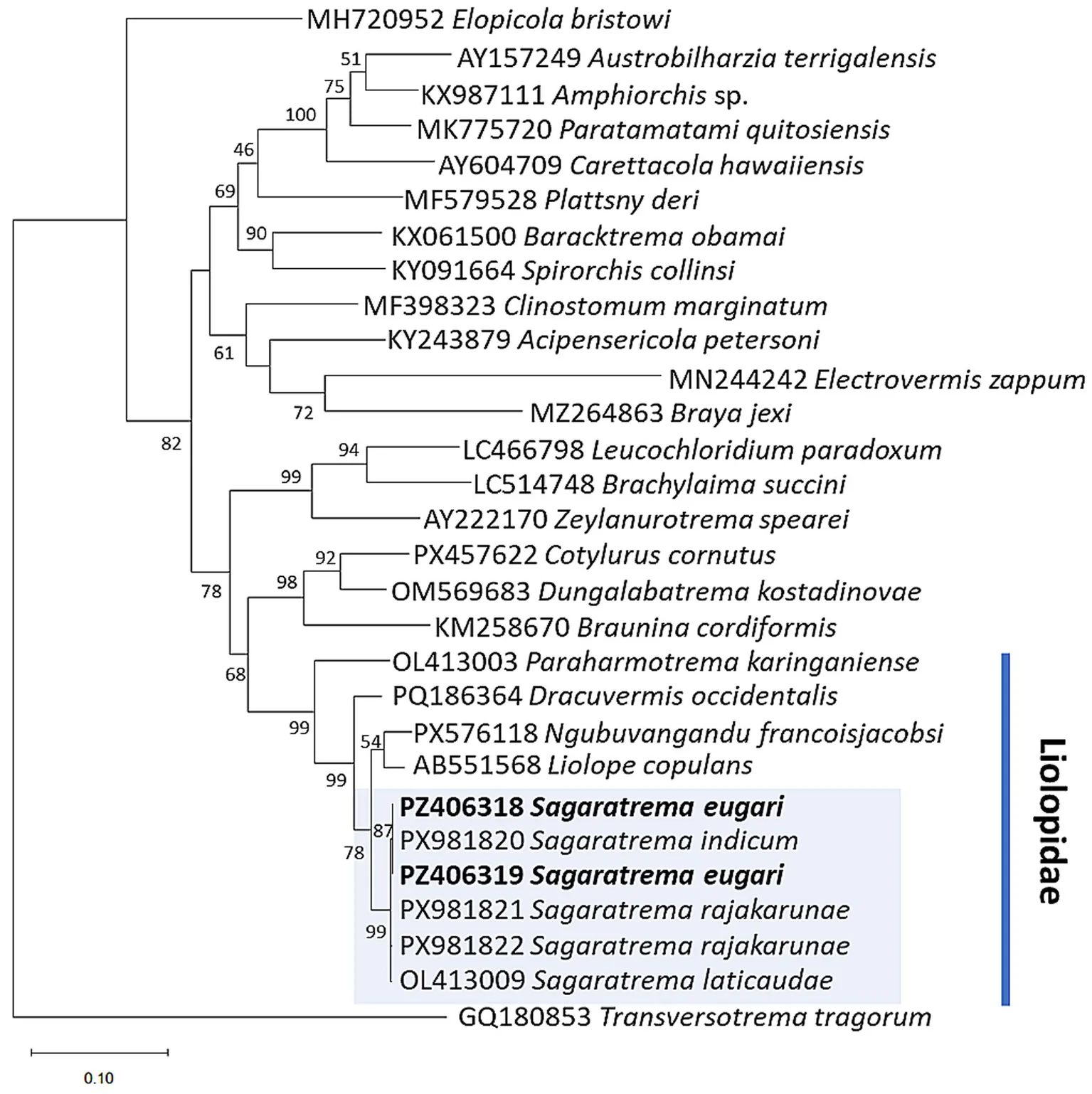
Phylogenetic relationships of Liolopidae species and related families within Diplostomida inferred from maximum likelihood analysis of 28S ribosomal DNA sequences. Newly generated sequences are shown in bold. Bootstrap support values below 40% are omitted. The scale bar represents the expected number of substitutions per site.

**Figure 3:**
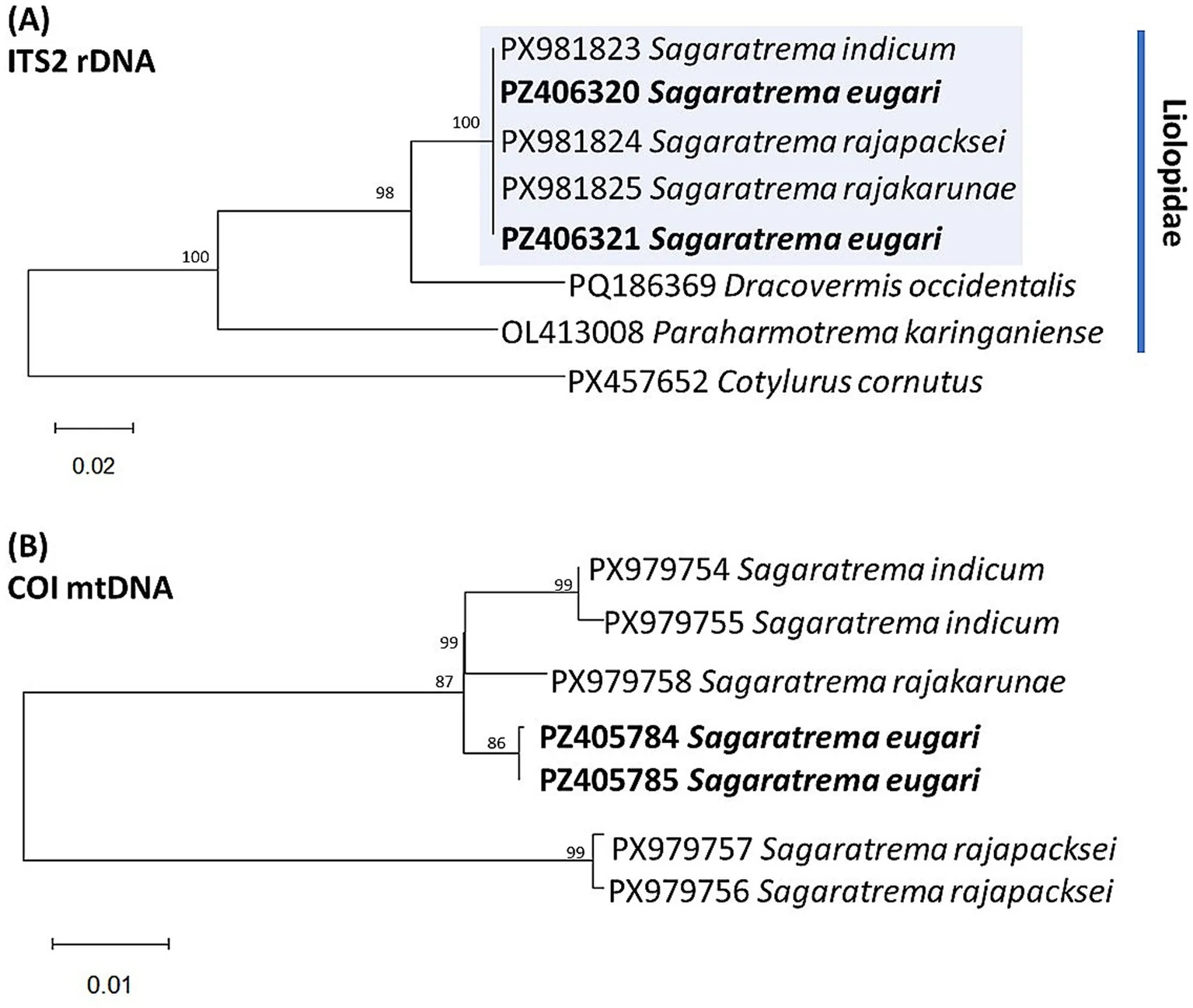
**(A)** Phylogenetic relationships of Liolopidae species inferred from maximum likelihood analysis based on ITS2 ribosomal DNA sequences. **(B)** Unrooted neighbour-joining tree based on *COI* mitochondrial DNA sequences. Newly generated sequences are shown in bold. The scale bar indicates the expected number of substitutions per site.

Phylogenetic analyses based on *28S* rDNA and ITS2 sequences showed that the specimens examined in the present study clustered within the well-supported clade of *Sagaratrema* (Figures 2, 3A) within the family Liolopidae, together with available sequences of *S. indicum*, *S. laticaudae*, *S. rajakarunae*, and *S. rajapaksei*. Due to the low genetic divergence among these taxa, species-level differentiation using rDNA markers was not possible. Genetic distances among ITS2 sequences of *Sagaratrema* spp. were 0.000. Genetic distances between *28S* sequences of species here studied and *S. indicum* was 0.0000, *S. rajakarunae* 0.0011–0.0022, and *S. laticaudae* 0.0022. The *18S* rDNA sequence generated in the present study was not included in the phylogenetic analyses because insufficient comparative sequences are currently available in public databases to allow robust phylogenetic inference. However, the unrooted Neighbor-Joining analysis based on *COI* sequences allowed *S. eugari* to be distinguished from the other available *COI* sequences, showing a closer relationship to *S. rajakarunae* and *S. indicum* (Figure 3B).

## 4 Discussion

According to our findings, the little file snake, *A. granulatus*, should be considered a definitive host of *S. eugari*. In the original description, the parasite was reported from the Philippine cobra, *N. philippinensis*; however, the presence of *A. granulatus* was recorded on Luzon Island, in the same area as the original description. The little file snake has also been cited from Mindanao Island, where *S. eugari* was recorded from the Southeast Asian bockadam, *C. rhynchops* (20). These overlapping distributions raise the possibility that *A. granulatus* may play a more important role in the life cycle of *S. eugari* than previously recognized. However, the available helminthological data remain insufficient to determine the relative importance of the different snake species reported as hosts.

If liolopids originally possessed freshwater life cycles, but some species of *Sagaratrema* now complete their development in marine environments, at least two evolutionary scenarios may explain this transition. One possibility is that ancestral *Sagaratrema* species colonized marine environments together with their snake hosts during the evolutionary shift from terrestrial to marine habitats. Such a transition would have required a corresponding change in intermediate hosts, from freshwater to marine gastropods, potentially through estuarine stages that facilitated adaptation to saline environments (21). Alternatively, the parasites may have initially switched to snake hosts occupying transitional coastal or estuarine habitats and only later adapted to fully marine intermediate hosts, implying that colonization of marine environments by the parasites did not necessarily occur simultaneously with that of their definitive hosts (2). Within this evolutionary framework, *S. eugari* remains unusual among congeners because it has been recorded from both terrestrial and semi-aquatic snakes rather than exclusively from hydrophiine sea snakes. These evolutionary scenarios remain speculative and require confirmation through future studies on the life cycles, host associations, and phylogenetic relationships of liolopid trematodes.

Although *A. granulatus* occupies coastal and estuarine habitats, its feeding ecology and phylogenetic distance from elapid snakes may limit its exposure to the trophic pathways involved in the life cycles of these parasites. In addition, the limited sampling across its biogeographic distribution and the relatively small number of hosts examined to date may have contributed to the absence of previous records of liolopids of this genus in *A. granulatus*. Clarifying the host range and transmission ecology of *Sagaratrema*, particularly for *S. eugari*, is therefore essential for understanding the evolutionary history of the group within the Liolopidae.

The morphology of our specimens showed that the vitellarium can reach or slightly surpass the ventral sucker. In the original description by Tubangui and Masiluñgan (3), this feature was not observed, probably because of the limited number of specimens examined. We also observed individuals in which the vitellarium does not reach the ventral sucker. This observation suggests that the anterior extent of the vitellarium may be more variable within *S. eugari* than previously recognized and should be interpreted with caution when comparing specimens. It should be noted that the vitellarium in the postesticular region is constrained to the ceca, not reaching the excretory system as in *S. indicum* (2).

On the other hand, the present study provides the first molecular data for *S. eugari*, complementing its morphological identification and helping to clarify the phylogenetic relationships within the genus *Sagaratrema* and the family Liolopidae. The study by De Silva et al. (2) highlighted *S. eugari* as a missing link within *Sagaratrema*, emphasizing two major gaps in knowledge: its phylogenetic position and its ecological associations. The present study addresses the first of these gaps and advances understanding of the species’ ecology by identifying *A. granulatus* as a definitive host. Because molecular data were generated from only two specimens, intraspecific genetic variation in *S. eugari* could not be adequately assessed. However, the identity and ecology of the intermediate hosts remain unresolved. The little file snake occupies a broad range of habitats, from mangroves to coastal marine environments, suggesting that transmission pathways involving intermediate hosts, presumably mollusks and fishes, remain unclear. Because *A. granulatus* moves across mangrove, estuarine, and marine habitats, it is difficult to determine where transmission occurs between the first and second intermediate hosts and subsequently to the definitive host. In conclusion, the present findings demonstrate that *Sagaratrema* is not restricted to elapid snakes as its principal definitive hosts and that its host range also includes *A. granulatus* (Acrochordidae).

## Data Availability

The datasets presented in this study can be found in online repositories. The names of the repository/repositories and accession number(s) can be found in the article/supplementary material.

## References

[1] Niewiadomska K. "Family Liolopidae Odhner, 1912". In: Gibson DI Jones A Bray RA, editors. Keys to the Trematoda. Wallingford: CABI Publishing (2002). p. 121–5.

[2] De Silva MLI Pathirana E Rajapakse J Martin S. Return to the sea: *Sagaratrema* n. g. for the marine Liolopidae (Digenea: Diplostomida) with two new species parasitic in snakes from Sri Lanka. Parasitology. (2026) 153:1–17. doi: 10.1017/S0031182026101802 PMC13315225 41776734

[3] Tubangui MA Masiluñgan VA. Trematode parasites of Philippines vertebrates-VIII. Flukes from a cobra and a crocodile. Philipp J Sci. (1936) 60:255–66.

[4] Fischthal JH Kuntz RE. Annotated record of some previously described digenetic trematodes of amphibians and reptiles from the Philippines, Korea, and Matsu Island. Proc Helminthol Soc Wash. (1967) 34:104–9.

[5] Bernstein JM Bautista JB Clores MA Brown RM Ruane S Sanguila MB . Using mangrove and field observation data to identify fine-scale species distributions: a case study in bockadams (Serpentes: Homalopsidae: *Cerberus*). R Soc Open Sci. (2024) 11:240483. doi: 10.1098/rsos.240483, 39469132 PMC11515136

[6] Adlard RD Barker SC Blair D Cribb TH. Comparison of the second internal transcribed spacer (ribosomal DNA) from populations and species of Fasciolidae (Digenea). Int J Parasitol. (1993) 23:423–5. doi: 10.1016/0020-7519(93)90022-Q, 8359995

[7] Olson PD Cribb TH Tkach VV Bray RA Littlewood DTJ. Phylogeny and classification of the Digenea (Platyhelminthes: Trematoda). Int J Parasitol. (2003) 33:733–55. doi: 10.1016/s0020-7519(03)00049-3, 12814653

[8] Gilardoni C Etchegoin J Cribb T Pina S Rodrigues P Diez ME . Cryptic speciation of the zoogonid digenean *Diphterostomum flavum* n. sp. demonstrated by morphological and molecular data. Parasite. (2020) 27:44. doi: 10.1051/parasite/2020040, 32553099 PMC7301638

[9] León-Règagnon V. Evidence of new species of *Haematoloechus* (Platyhelminthes: Digenea) using partial cox1 sequences. Mitochondrial DNA. (2010) 21:12–7. doi: 10.3109/19401736.2010.523700, 21271853

[10] Sanders KL Hamidy A Head J Gower DJ. Phylogeny and divergence times of filesnakes (*Acrochordus*): inferences from morphology, fossils and three molecular loci. Mol Phylogenet Evol. (2010) 56:857–67. doi: 10.1016/j.ympev.2010.04.031 20434568

[11] Chattopadhyaya DR. Studies on the trematode parasites of reptiles found in India. (digenetic flukes from the marine snakes of the coast of Bombay). Helminthologia. (1970) 11:35–62.

[12] Jones HI. Gastrointestinal nematodes from aquatic Australian snakes. Mem Qd Mus. (1978) 18:243–63. doi: 10.3316/informit.T2025102000001200443390384

[13] Suwannarat N Gilardoni C Ribas A Miquel J Poonlaphdecha S. Molecular characterization of three digeneans parasitizing marine fish in the Gulf of Thailand. Front Vet Sci. (2025) 12:1694904. doi: 10.3389/fvets.2025.1694904, 41502855 PMC12769086

[14] Cremonte F Gilardoni C Pina S Rodrigues P Ituarte C. Revision of the family Gymnophallidae Odhner, 1905 (Digenea) based on morphological and molecular data. Parasitol Int. (2015) 64:202–10. doi: 10.1016/j.parint.2014.12.003, 25510312

[15] Wee N Cribb TH Bray RA Cutmore SC. Two known and one new species of *Proctoeces* from Australian teleosts: variable host-specificity for closely related species identified through multi-locus molecular data. Parasitol Int. (2017) 66:16–26. doi: 10.1016/j.paint.2016.11.008, 27871817

[16] Ivanova NV Borisenko AV Hebert PDN. Express barcodes: racing from specimen to identification. Mol Ecol Resour. (2009) 9:35–41. doi: 10.1111/j.1755-0998.2009.02630.x, 21564962

[17] Kumar S Stecher G Li M Knyaz C Tamura K. MEGA X: molecular evolutionary genetics analysis across computing platforms. Mol Biol Evol. (2018) 35:1547–9. doi: 10.1093/molbev/msy096, 29722887 PMC5967553

[18] Darriba D Taboada GL Doallo R Posada D. JModelTest 2: more models, new heuristics and parallel computing. Nat Methods. (2012) 9:772. doi: 10.1038/nmeth.2109 PMC4594756 22847109

[19] Hoang DT Chernomor O Von Haeseler A Minh BQ Vinh LS. UFBoot2: improving the ultrafast bootstrap approximation. Mol Biol Evol. (2018) 35:518–22. doi: 10.1093/molbev/msx281, 29077904 PMC5850222

[20] GBIF Secretariat GBIF Backbone Taxonomy [Checklist dataset] (2023)

[21] Heatwole H Grech A Marsh H. Paleoclimatology, paleogeography, and the evolution and distribution of sea kraits (Serpentes; Elapidae; *Laticauda*). Herpetol Monogr. (2017) 31:1–17. doi: 10.1655/HERPMONOGRAPHS-D-16-00003

[22] Dutton HR DuPreez LH Urabe M Bullard SA. *Paraharmotrema karinganiense* n. gen., n. sp. (Digenea: Liolopidae) infecting the intestine of serrated hinged terrapin (*Pelusios sinuatus*), east African black mud turtle (*Pelusios subniger*), and south African helmeted turtle (*Pelomedusa galeata*) and a phylogenetic hypothesis for liolopid genera. Int J Parasites Wildl. (2022) 17:43–52. doi: 10.1016/j.ijppaw.2021.09.006, 34976724 PMC8688888

[23] Baba T Hosoi M Urabe M Shimazu T Tochimoto T Hasegawa H. *Liolope copulans* (Trematoda: Digenea: Liolopidae) parasitic in *Andrias japonicus* (Amphibia: Caudata: Cryptobranchidae) in Japan: life cycle and systematic position inferred from morphological and molecular evidence. Parasitol Int. (2011) 60:181–92. doi: 10.1016/j.parint.2011.02.002, 21345377

[24] Dutton HR Bullard SA Brule JH Kelly AM. Redescription of *Dracovermis occidentalis* (Digenea: Liolopidae) infecting American alligator, *Alligator mississippiensis* from the bon-Secour River (Mobile–Tensaw River Delta, Alabama, USA) and a revised phylogeny for Liolopidae. Parasitol Res. (2024) 123:326. doi: 10.1007/s00436-024-08339-2, 39297894 PMC11413115

[25] Dutton HR Jacobs FJ Beytell PC Netherlands EC DuPreez LH Bullard SA. New genus and species of liolopidae Odhner, 1912 (Platyhelminthes: Digenea) infecting Nile crocodile, *Crocodylus niloticus* (Laurenti, 1768) (Crocodilia: Crocodylidae) in the Kavango River, Namibia. J Parasitol. (2026) 112:21–7. doi: 10.1645/25-36 41592706

[26] Stanevičiūtė G Stunžėnas V Petkevičiūtė R. Morphological and molecular studies of tetracotyle-type metacercariae of the genus *Cotylurus* Szidat, 1928 (Trematoda) from the gravel snail *Lithoglyphus naticoides* (Gastropoda) and host sex dependent differences in infection rate. Pathogens. (2025) 14:1063. doi: 10.3390/pathogens14101063, 41156671 PMC12567010

